# Impact of Non-Pharmacological Interventions on the Mechanisms of Atherosclerosis

**DOI:** 10.3390/ijms23169097

**Published:** 2022-08-13

**Authors:** Daniela Matei, Ioana Buculei, Catalina Luca, Calin-Petru Corciova, Doru Andritoi, Robert Fuior, Daniel-Andrei Iordan, Ilie Onu

**Affiliations:** 1 Department of Biomedical Sciences, Faculty of Medical Bioengineering, University of Medicine and Pharmacy “Grigore T. Popa” Iasi, 700454 Iasi, Romania; 2 Doctoral School of the Faculty of Medicine, University of Medicine and Pharmacy “Grigore T. Popa” Iasi, 700454 Iasi, Romania; 3 Faculty of Electrical Engineering, “Gheorghe Asachi” Technical University of Iasi-Romania, 21-23 Prof. D. Mangeron Blvd., 700050 Iasi, Romania; 4 Department of Individual Sports and Kinetotherapy, Faculty of Physical Education and Sport, “Dunărea de Jos” University of Galati, 800008 Galati, Romania; 5 Center of Physical Therapy and Rehabilitation, “Lower Danube” University of Galati, 800008 Galati, Romania; 6 Doctoral School of Faculty of Chemical Engineering and Environmental Protection “Cristofor Simionescu”, Technical University “Gheorghe Asachi” Iasi, 700050 Iasi, Romania

**Keywords:** endothelial dysfunction, sympathetic nervous system, caloric restriction, fasting, diet, physical exercise, vagus nerve stimulation

## Abstract

Atherosclerosis remains the leading cause of mortality and morbidity worldwide characterized by the deposition of lipids and fibrous elements in the form of atheroma plaques in vascular areas which are hemodynamically overloaded. The global burden of atherosclerotic cardiovascular disease is steadily increasing and is considered the largest known non-infectious pandemic. The management of atherosclerotic cardiovascular disease is increasing the cost of health care worldwide, which is a concern for researchers and physicians and has caused them to strive to find effective long-term strategies to improve the efficiency of treatments by managing conventional risk factors. Primary prevention of atherosclerotic cardiovascular disease is the preferred method to reduce cardiovascular risk. Fasting, a Mediterranean diet, and caloric restriction can be considered useful clinical tools. The protective impact of physical exercise over the cardiovascular system has been studied in recent years with the intention of explaining the mechanisms involved; the increase in heat shock proteins, antioxidant enzymes and regulators of cardiac myocyte proliferation concentration seem to be the molecular and biochemical shifts that are involved. Developing new therapeutic strategies such as vagus nerve stimulation, either to prevent or slow the disease’s onset and progression, will surely have a profound effect on the lives of millions of people.

## 1. Introduction

The global burden of atherosclerotic cardiovascular disease is steadily increasing and it is considered the largest known non-infectious pandemic [1]. Atherosclerosis is a vascular disease, characterized by the thickening and hardening of the arterial wall with the progressive reduction in the lumen and the generation of ischemic clinical signs in the respective territories.

Atherosclerosis remains the leading cause of mortality and morbidity worldwide [1]. It is characterized by the deposition of lipids and fibrous elements in the form of atheroma plaques, in vascular areas which are hemodynamic overloaded. It mainly affects the elastic and muscular arteries, of large and medium caliber, with the most affected being the coronary and cerebral arteries.

Atherosclerosis develops gradually, over several decades, following a discontinuous rate with periods of stagnation and rapid evolution. Unchangeable constitutional factors include age, sex and genetic factors, while modifiable risk factors such as hyperlipidemia, hypertension, smoking, diabetes and physical inactivity are the target of the main studies on preventing and combating the formation of atherosclerotic plaques.

## 2. The Mechanism of Atherosclerotic Plaques Formation

The endothelium is not only a barrier between the blood and the tissues, but is also an important autocrine and paracrine organ that regulates vascular tone, cellular composition of the vascular wall, leucocyte trafficking, blood fluidity, inflammation, angiogenesis, and other processes [2]. Endothelium is of crucial importance for internal homeostasis, and it is considered a “first line” physiological defense against atherosclerosis [3,4].

Endothelium can regulate vascular tone through endothelium-derived contracting factors (endothelin, prostaglandin F2a and thromboxane A2) and endothelium-derived relaxing factor (prostaglandin I2 and nitric oxide) [5,6]. These substances can directly act on vascular smooth muscle cells (VSMCs) or by affecting sympathetic activity [7]. Endothelium-derived relaxing factor (EDRF) is stimulated by acetylcholine. While nitric oxide (NO) has mainly been implicated in the regulation of larger vessels, other vasodilators seem to play an important role in microcirculation [8,9,10,11]. Other substances, such as angiotensin, insulin, natriuretic peptide, adiponectin, uric acid, lipids, reactive oxygen and nitrogen species, can alter the endothelium function [7].

In normal endothelium, there is a balance between vasoconstrictors and vasodilators factors. When this balance is altered, endothelial dysfunction occurs, which is a key initiating event in atherosclerosis. Endothelial dysfunction predisposes the vasculature to vasoconstriction; leukocyte adherence, platelet activation, mitogenesis, pro-oxidation, thrombosis, impaired coagulation, vascular inflammation, and all these processes lead to atherosclerosis.

Nitric oxide is synthesized from L-arginine under the influence of the three isoforms of NO synthase, such as neuronal NOS (nNOS), inducible NOS (iNOS) and endothelial NOS (eNOS). Endothelial NOS is stimulated by estrogens, insulin, glucagon-like peptide, thyroid hormones, erythropoietin, and high-density lipoproteins, dietary factors, and repetitive increase in shear stress, as occurs in exercise, and all these factors increase NO bioavailability [12,13]. On the other hand, the factors that negatively impact eNOS are reactive oxygen species (ROS), reduced availability of arginine, increased levels of arginase, asymmetric dimethylarginine (ADMA), cortisol, aldosterone, smoking, hyperglycemia, insulin resistance, hypercholesterolemia and obesity, homocysteinemia, and uric acid [12].

NO relaxes VSMCs, produces vasodilatation, decreases peripheral resistance, inhibits the proliferation and migration of vascular smooth muscle cells, prevents vascular remodeling, and inhibits monocytes migration and platelet adhesion/aggregation. Additionally, NO has antioxidant actions and anti-atherosclerotic effects. NO can modulate sympathetic neurotransmission, modifying the vascular smooth muscle tone, in various vascular beds [14,15,16].

NO inhibits the oxidation of LDL cholesterol, which plays a major role in initiating the process of atherosclerosis. Endothelial dysfunction decreases the synthesis and release of nitric oxide, which causes vasoconstriction, inflammation, platelet aggregation, the proliferation and migration of VSMCs and oxidative stress.

Hyperglycemia, insulin resistance and the excess release of free fatty acids from adipose tissue lead to the activation of protein kinase C, inhibition of phosphatidylinositol-3-kinase (PI-3) and increase in ROS, mechanisms that directly alter the production of NO or reduce the bioavailability of the nitric oxide that was already produced [17,18]. NO has been viewed as a vasodilator substance but NO can also interplay with adrenergic pathways and increase noradrenaline release, which will produce vasoconstriction.

In endothelial dysfunction, an increase in the serum level of endothelin (ET-1), a powerful vasoconstrictor, can be observed. The synthesis of ET-1 is stimulated by angiotensin II, norepinephrine, glucocorticoids, hypoxia, shear stress, lipoproteins, oxidative stress and by acute mental or physical stress [19,20]. In the nervous system ET-1 plays a role in the cardiovascular sympathetic response to stress [21]. ET-1 also produces fibrosis of the vascular wall, stimulation of vascular smooth muscle, increased ROS and has a pro-inflammatory effect [22]. ET-1 contributes to the activation of transcription factor-κB (NF-κB), and stimulates the production of pro-inflammatory cytokines like, TNFα, IL-1, and IL-6 [23].

In laminar flow, endothelial cells express antithrombotic, anti-inflammatory, and antioxidant proteins, such as eNOS, cyclooxygenase- 2 (COX-2), and manganese-dependent superoxide dismutase (SOD) [24]. In turbulent flow, NF-κB pathways are activated, promoting an inflammatory pattern. Additionally, zinc finger transcription factor Kruppel-like factor 2 (KLF2) and nuclear factor erythroid-2-related factor-2 (Nrf2), were identified by comparing endothelial gene expressions under different hemodynamic patterns [25]. KLF-2 can inhibit inflammatory factors and restore NO levels. KLF-2 blocks IL-1β and inhibits vascular cell adhesion molecules-1 (VCAM-1) and E-selectin expression to disturb the adhesion of immune cells [26]. Nrf2 is responsible for regulating redox-related genes (heme oxygenase 1, ferritin heavy chain, NADPH dehydrogenase quinone 1, and thioredoxin reductase) to maintain vascular redox balance in laminar flow [25,26].

The initial event for atherosclerosis development is endothelial dysfunction, which is favored by the disturbance of lipid homeostasis. Higher plasma lipid levels with smaller lipoproteins, such as low-density lipoproteins (LDL), small dense LDL and triglyceride, accumulate in the intima and activate the endothelium [27]. In areas of hemodynamic turbulence, monocytes’ adhesion to the endothelium is favored by stimulating VCAM-1, as well as the secretion of interleukin-1b and monocyte-colony-stimulating factor (MCSF) [27].

Proatherogenic-modified low-density lipoprotein (mLDL) plays an important role in the accumulation of cholesterol and lipids in the arterial wall. These modified lipoproteins bind with proteoglycans of the extracellular matrix in the intima of blood vessels, causing the aggregation of lipoprotein particles, foam cell formation, endothelial destruction, leukocyte recruitment and inflammation [28]. Thereafter, an inflammatory response in the artery wall occurs, followed by mononuclear adhesion and migration into the arterial subendothelial space [27,28].

Studies conducted in this field show that inflammation is highly related to the initiation and progression of atherosclerotic plaque, and inflammatory factors are present throughout its pathogenesis. In the early stages of atherosclerosis, inflammation causes a change in the endothelial cells, and in the late stages of atherosclerosis, the matrix metalloproteinases (MMPs) produced by inflammatory cytokines that infiltrate the vessel wall will degrade the collagen fibers of the extracellular matrix of the plaque, which can cause plaque rupture, bleeding, and thrombosis. CRP and adhesion molecules IL-6 and MMPs are inflammatory biomarkers that can be used in the diagnosis of atherosclerosis and cardiovascular disease. Toll-like receptor 4, NF-κB, and JAK/STAT are atherosclerosis-related inflammatory signaling pathways [29].

Oxidation of LDL (ox-LDL) increases inflammation, VSMCs proliferation, and macrophage activation [30]. Monocytes engulf ox-LDL, differentiate into macrophages and foam cells, in the arterial sub-endothelial space that generate reactive oxygen and nitrogen species (ROS, RNS), proteases, lipases and express scavenger receptors and toll-like receptors at a high level [31]. Scavenger receptors may contribute to the oxidized LDL particles. Lipid depositions into macrophages contribute to foam cell formation and the appearance of lipid striae. Therefore, oxidized LDL may play a role in impairing endothelial function damage and initiating the atherosclerosis process [32,33]. Toll-like receptors can stimulate the production of inflammatory cytokines, proteases, and cytotoxic radical molecules [34].

Plasma lipoproteins, low- and high-density lipoprotein, are synthesized in the liver. The synthesis of cholesterol occurs in the endoplasmic reticulum or can be obtained via circulation through the low-density lipoprotein receptor (LDLR) [35].

Cholesterol is synthesized from acetyl-CoA under the action of the enzyme 3-hydroxy-3-methylglutaryl coenzyme A reductase (HMGCR), which catalyzes the conversion of HMG-CoA to mevalonic acid [35]. After receptor binding, internalization occurs and then it is transported to the late endosomal/lysosomal system, where LDL-cholesterol esters are hydrolyzed by acid lipase [36]. The expressions of the LDLR are regulated by the two transcription factor families: the liver X receptors (LXRs) and sterol regulatory element-binding proteins (SREBPs) [37,38].

When intracellular levels of cholesterol are high, ox-LDL activates LXRs to induce the expression of genes that reduce intracellular cholesterol [39]. If cholesterol levels decline, SREBPs induce the expression of HMGCR and LDLR, thereby increasing cholesterol biosynthesis. Decreases in MMAB, a SREBP2-target gene that catalyzes the conversion of vitamin B12 to adenosylcobalamin, inhibit HMGCR activity and cholesterol biosynthesis [40], increasing SREBP2-mediated gene expression and LDL-C uptake. Hepatic levels of MMAB are modulated by dietary and cellular cholesterol levels through SREBP2 [40].

Biologically active cholesterol metabolites, such as desmosterol, lanosterol, and 25-hydroxycholesterol (25-HC), can regulate immune response in conditions of lipid overloading and activate the innate immune system [41,42,43]. Desmosterol is most commonly found in human atherosclerotic coronary artery plaques and is a major endogenous liver X receptor (LXR) ligand, involved in the LXR/retinoid X receptor (RXR) activation and thus macrophage foam cell formation.

The overexpression of 3β-hydroxysterol Δ24-reductase (DHCR24), the enzyme that catalyzes the conversion of desmosterol to cholesterol, causes desmosterol depletion from the cells [44]. Decreased desmosterol levels promote the generation of ROS, increase interferon responses, and reduce the expression of anti-inflammatory macrophage markers. A recent study using a generated transgenic mouse model highlights that desmosterol deficiency in macrophages contributes to impaired liver X receptor (LXR) activation, lipid accumulation, mitochondrial metabolic alterations, and enhanced inflammasome activity [44]. LXRs produces gene expression within the efflux pathway, including ATP-binding cassette (ABC) proteins A1 and G1 (ABCA1/G1), which are cellular cholesterol transporters [45]. LXR also controls the expression of the inducible degrader of the low-density lipoprotein receptor (IDOL), which promotes the degradation of LDLR [45]. The repression of ABCA1 is considered the primary mechanism by which miR-33, microRNA encoded in SREBP’s intronic region, regulates macrophage cholesterol efflux and atherogenesis [46]. High-density lipoprotein cholesterol (HDL-C) confers atheroprotection through the reverse cholesterol transport (RCT) pathway [47]. RCT begins with the removal of cholesterol from foam cells, which can be derived from monocytes but can also be macrophage-like cells originating from cholesterol-laden vascular smooth muscle cells [48]. This pathway enables free cholesterol efflux to cholesterol acceptors, such as nascent or mature HDL, together with the exit of macrophages from plaques. HDL acts as the specific cholesterol acceptor that transports excess cholesterol from peripheral tissue to the plasma, and then delivers it to the liver, either directly, via binding to the hepatic HDL receptor SR-B1, or indirectly, via apoB-containing lipoproteins. The last step of the RCT is cholesterol excretion into the feces through biliary cholesterol excretion or trans-intestinal cholesterol efflux [49]. In addition to mediating RCT by serving as a cholesterol acceptor, HDL is also known to have antioxidant and anti-inflammatory effects that can improve RCT and plaque regression [50]. Many HDL subtypes are recognized, which are classified according to their physicochemical properties: density (HDL2, HDL3), protein (apoA-I, A-II or both), form, size, etc. [51]. The RTC is linked to apoA-I. Macrophage RTC may stimulate the cholesterol efflux and may also involve cholesterol removal from plaques by another mechanism, namely the emigration of the macrophages themselves [52].

The immune responses in atherosclerosis include both innate and adaptive responses [53]. During chronic inflammation, at the site of atherosclerosis-diseased adventitia, a tertiary lymphoid organ occurs, due to the accumulation of immune cells through the involvement of lymphoid tissue-organizer-like cells [54]. At the site of the inflammatory process, several adhesion molecules and chemokines promote monocyte recruitment, as well as the attraction of T cells. Moreover, plaque macrophages and T cells secrete pro-inflammatory cytokines, such as interferon-γ, interleukin-2 (IL-2), tumor necrosis factor-α, and -β, as well as anti-inflammatory cytokines such as IL-10 and IL-4 [55]. In this way, the inflammatory process can stop or continue depending on the type of cytokine that predominates. The artery tertiary lymphoid organ (ATLOs) has the same structure as the secondary lymphoid organs, including T cell areas and B cell follicles [56]. ATLO development occurs in three stages. In the first stage, there are few T-type and B-type cells. In the second stage, the appearance of follicular dendritic cells is not observed, but the number of T-type and B-type cells is increased in a separated area [54]. In the third stage, ATLO contains separate B cell follicles with follicular dendritic cells in activated germinal centers; T cell areas mainly contain T cells, Treg cells, DCs, and macrophages [54]. Since ATLOs contain both pro- and anti-inflammatory immune cells, the inflammatory process in atherosclerosis can be stopped if interventions occur in time. ATLOs that form in the adventitia during atherosclerosis can regulate the immune response and may modulate RCT flux [57]. The lymphatic vasculature, in addition to the drainage of inflammatory cells and cytokines, is important for the removal of cholesterol from macrophages in RCT, accounting for 50% of cholesterol delivery from cholesterol-loaded macrophages into the plasma compartment [58]. Moreover, lymphatic insufficiency in mice disrupts proper lipoprotein metabolism, leading to accelerated atherosclerosis [59].

Vessels contain sympathetic nerves, which are distributed between smooth muscle and adventitia layers [60]. At present, it is well established that the sympathetic nervous system (SNS) contributes to the modulation of vascular function and this relationship is a key factor in the development of cardiovascular diseases. Several factors, such as the renin–angiotensin system, NO, ROS and endothelin, influence this modulation at central and peripheral levels [61,62,63]. The parasympathetic nervous system (PNS) does not directly act on the blood vessels, the necessary parasympathetic endings missing from this level.

In the adventitia layer, which is very close to ATLOs, expanded nerve ending networks were highlighted. Researchers have introduced a neurotropic virus into the adventitia of mice and tracked its path to the central nervous system [64]. They were able to establish a structural artery–brain circuit (ABC): nociceptive afferents from abdominal adventitia entered the central nervous system through spinal cord T6–T13 dorsal root ganglia and reached structures such as the parabrachial nucleus, central amygdala [64]. Sympathetic efferent fibers start from these structures, projected from medullary and hypothalamic neurons to the adventitia through spinal intermediolateral neurons and both coeliac and sympathetic chain ganglia [64]. The increased activity of sympathetic nerve fibers increases the growth of atheromas, whereas coeliac ganglionectomy reduced disease progression and enhanced plaque stability. Additionally, the neurotransmitter norepinephrine released (NE) is selectively up-regulated in the adventitia of atherosclerotic segments [65].

The SNS contributes to the differentiation, maturation, recruitment, and regulation of immune cells via lymphoid organs [66] and also influences vascular and lymphatic flow [67]. Cytokines such as interleukin-1, interleukin-6, and tumor necrosis factor-alpha, ROS, RNS can stimulate SNS. Even if there are no necessary terminations in the adventitia of the vessels, the PNS innervates the cells of the immune system.

The efferent parasympathetic nerves directly release acetylcholine or instruct immune cells to produce acetylcholine at the site of inflammation, which inhibits pro-inflammatory cytokine release [68]. This inhibitory pathway mediated by parasympathetic vagus nerve is called the cholinergic anti-inflammatory pathway [68,69]. The components of this neuro-immune circuit include the vagus nerve, the splenic nerve, choline acetyltransferase expressing Tcells (TChAT), and the α7 nicotinic acetylcholine receptor subunit (α7nAChR) [70,71,72].

The α7nAChR is reportedly expressed in human atherosclerotic lesions and their ablation increased aortic atherosclerosis [73]. However, the activation of α7nAChR reduces inflammation in a wide range of animal models of different inflammatory diseases [74].

Acetylcholine (ACh), a neurotransmitter of the vagus nerve, is biosynthesized by ChAT and is an agonist for α7nAChR. ACh can also be released to the extracellular space by TChAT, which relays neural signals to α7nAChR-expressing macrophages in spleen and attenuates the production of pro-inflammatory cytokines [70,72]. Galantamine administration, an acetylcholinesterase inhibitor, decreases pro-inflammatory cytokine levels and improves insulin sensitivity [75]. Cholinergic agonists and vagus nerve stimulation blocked endothelial cell activation and the recruitment of immune cells to sites of inflammation [76].

Due to the pro-inflammatory and oxidizing elements, the differentiation of VSMCs changes from a contractile to an activated secretory and migratory phenotype [77,78]. Activated VSMCs migrate from the media into the intima, increasing collagen and elastin deposition and creating a fibrous capsule, which includes extracellular lipid material and cellular debris. This process contributes to the thickening of atherosclerosis plaques and the narrowing of the vascular lumen.

The main chemotactic and mitogenic factors are platelet-derived-growth factors, produced by platelets, endothelial cells, macrophages, vascular smooth muscle cells, IL -1, IL-6, thrombin, TNF-alpha, and INF-gamma. Proliferated muscle cells acquire macrophage-like properties and secretory properties, producing collagen fibers and proteoglycans that form a fibrous capsule that includes extracellular lipid material and cellular debris.

Fibrous caps may rupture, exposing the underlying extremely thrombogenic core. The terminal phase of atherosclerosis is associated with the rupture of plaques, especially those with a higher lipid deposition. The rupture of the plaque can be superficial, when only the denudation of the endothelium takes place or it can be deep, when the rupture includes the whole collagen layer, reaching the atherosclerosis deposit. The blood enters the plaque and contacts thromboplastin factors, activating platelets and the coagulation system. Increased thrombotic factors, such as von Willebrand factor and thromboxane A2, and decreased amounts of antithrombotic factors (e.g., heparin), lead to clot formation and enlargement [79]. Initially, an intramural microthromb is formed; sometimes, it develops very quickly in the lumen and life-threatening complications such as infarction are linked to plaque erosion or rupture, while sometimes this occurs without previous symptomatic stenosis.

## 3. Non-Pharmacological Options in Atherosclerosis

The management of atherosclerotic cardiovascular disease increases the cost of health care worldwide, which is a concern for researchers and physicians, leading to the search for effective long-term strategies to improve the efficiency of treatments by managing conventional risk factors.

The primary prevention of atherosclerotic cardiovascular disease is the preferred method to reduce cardiovascular risk. This is achieved by decreasing the prevalence of obesity, which is seen as the most urgent problem as it affects lipid profile, glucose metabolism, inflammation, and disease progression. A major obstacle to successful primary intervention is that atherosclerosis begins in early childhood and seamlessly progresses throughout an individual’s life, suggesting that childhood risk factors are similar to adult risk factors [80].

Atherosclerotic cardiovascular disease is the leading cause of death and disability. Prolonged survival with chronic disease explains why the prevalence, burden, and costs of the disease remain high [81]. Given these issues, aggressive treatment should be started at the first indication and continued over several years, along with the reduction in disease risk factors such as visceral adiposity, dyslipidemia, hypertension, and diabetes.

Low-risk lifestyle factors are known to reduce the risk of cardiovascular disease in a multifactorial manner, with a favorable prognosis. However, a negative prognosis is given by risk factors associated with an unhealthy lifestyle, such as poor diet, sedentary lifestyle, air pollution, lack of sleep, and psychosocial stress [82,83]. High-risk lifestyle factors cause phenotypic adaptations by maintaining a pro-inflammatory environment that favors atherosclerosis, and a low-risk lifestyle changes the interaction between tissues from a pro-inflammatory to an anti-inflammatory environment [84].

From this perspective, reducing the risk factors, along ensuring with a balanced lifestyle, is a powerful public health message for both clinicians and patients. The following lifestyle changes can help prevent or decrease the progression of atherosclerosis: quitting smoking, constant physical exercise, weight loss, calorie restriction or fasting, and stress management.

### 3.1. Caloric Restriction

Caloric restriction (CR) is a concept involving dietary interventions with chronic or periodic reduced energy intake, without malnutrition. CR prolongs a healthy lifespan in a variety of animal species and humans, but the most important limitation for humans is its long-term sustainability. CR generates cellular and metabolic adaptations that delay aging processes, prolong the maximum lifespan and consistently improve insulin resistance. Insulin resistance is associated with cardiovascular disease, but there are not enough data at present to show that increased insulin sensitivity reduces the progression of atherosclerosis [85,86].

Luigi Fontana et al. studied the long-term effects of CR by evaluating the atherosclerosis risk factors in people who restrict dietary intake with the aim of slowing aging. To demonstrate the long-term effects of CR, they evaluated the risk factors for atherosclerosis in people who restrict dietary intake to slow aging. The study was conducted on 18 individuals who followed CR for 6 years on average and another 18 healthy individuals who followed typical American diets. Carotid artery intima-media thickness, body composition, platelet-derived growth factor AB (PDGF-AB), CRP, blood pressure, serum lipids and lipoproteins, fasting plasma glucose, and insulin were assessed. Individuals in the CR group weighed less, BMI 19.6 ± 1.9 (body fat percentage 8.7 ± 7%), than the comparison group BMI 25.9 ± 3.2 kg/m^2^ (body fat percentage 24 ± 8%). Serum total cholesterol, low-density lipoprotein cholesterol, and the ratio of serum total cholesterol to high-density lipoprotein cholesterol (HDL-C) were lower in the CR group. Triglycerides, CRP, PDFG-AB, systolic and diastolic BP, fasting glucose, and fasting insulin were all markedly lower in the CR group, while HDL-C was higher, than in the American diet group. Carotid artery intima-media thickness was <40% lower in the CR group than in the comparison group. They concluded that long-term CR has a strong protective effect against atherosclerosis on healthy individuals [87].

CR is considered the most effective and reproducible dietary intervention, and is known to affect the aging process and increase healthy lifespan in primates and rodents. CR involves a 20–40% reduction in dietary requirements compared to normal intake; exceeding a 40% reduction can be considered a severe intervention, resulting in both beneficial and detrimental effects. However, there is no common agreement regarding how severe CR needs to be to have measurable benefits in different organs and systems [87,88].

CR improves risk factors for cardiometabolic disease, risks that include metabolic syndrome, atherogenic dyslipidemia (elevated triglycerides and fasting glucose, reduced high-density lipoprotein (HDL)-cholesterol), as well as high blood pressure and diabetes, and a large waist circumference. Visceral and subcutaneous adiposity leading to prediabetes and type 2 diabetes are seen as the most impotent risk factors in cardiometabolic diseases. CR can also enhance risk factors by imposing a negative energy balance-in both animal models and humans [86,89,90].

Mingyi Wang et al. published a study in 2018, conducted on 344 Fischer rats aged 6 months (young) and 24 months (old), which were divided into two heterogeneous groups. In the first group, they were fed ad libitum, and in the second group, from 1 month of age, they maintained 40% CR. The aortas were harvested, and a histopathological and morphometric analysis of the arterial wall was performed. The results demonstrated that CR significantly reduced age-associated intimal medial thickening, collagen deposition, and elastin degradation in arterial walls, which was attributable to platelet-derived growth factor signaling. CR contributes to the postponement of biological aging and the preservation of a younger aortic wall phenotype by limiting platelet-derived growth factor-associated signaling. The authors of this study conclude that CR, from a clinical perspective, has the following key elements: (i) CR increases the duration of vascular health by reducing age-associated proinflammation in the arterial wall in rodent models; (ii) CR effectively attenuates age-associated changes in the arterial proinflammatory phenotype at the molecular, cellular, and tissue levels; (iii) CR preserves a youthful arterial phenotype in old rodents [91].

The Comprehensive Assessment of Long-term Effects of Reducing Intake of Energy (CALERIE) was a two-year multicenter RCT to assess CR in healthy non-obese individuals for anti-aging adaptations in resting metabolic rate and core body temperature. CR reduced body weight, BMI, and blood pressure and improved plasma cholesterol concentrations to 300 kcal/day. At this resting metabolic rate, there was no significant influence on baseline body temperature. CALERIE answered a number of questions on CR in metabolically normal individuals by pointing out that there are effects on emerging measures of cardiometabolic risk and that these effects were modified by beginning BMI strata and gender [92,93].

William T. et al. conducted a study on 32 adult cynomolgus monkeys over a 4-year period to assess the impact of increased insulin sensitivity secondary to CR on atherosclerosis expansion. The primates were randomly assigned to a control group, fed a moderately atherogenic diet with 25 mg cholesterol/Cal containing 30% of calories from fat, while the CR group had a 30% reduction in total dietary intake, but cholesterol was maintained at the same value as the control group. In the CR group, significant improvements were observed in insulin sensitivity and reduced intra-abdominal fat, even though cholesterol intake concentration was similar in the two groups. Although significant improvements in insulin sensitivity were observed in the CR group, the extent of atherosclerosis did not differ between the two groups. Chronic CR is effective in reducing body fat and improving insulin sensitivity in aging. However, an increase in insulin sensitivity was not associated with a decrease in the extent of atherosclerosis, as LDL is the main risk factor for atherosclerosis, and high plasma cholesterol levels will cancel out the effects that were previously achieved by CR. In conclusion, CR prolongs maximum lifespan and delays aging processes, consistently improving insulin resistance in primates [94].

It is well established that CR delays aging processes, especially in primates. The mechanisms by which CR exerts its effects are not fully elucidated, but chronic CR is associated with improved insulin resistance, delaying physiological changes, and age-associated diseases. The molecular etiology of insulin resistance directly contributes to the development of atherosclerotic cardiovascular disease through the inhibition of nitric oxide production and stimulation of the Mitogen-Activated Protein Kinase (MAPK) pathway [95]. It has been shown that by using pharmacological or non-pharmacological approaches, such as CR, insulin resistance can be improved, with a positive impact on specific risk factors for cardiovascular disease in humans. Furthermore, it has been hypothesized that improving insulin sensitivity will lead to a reduction in cardiovascular events, which ultimately attenuates the progression of atherosclerosis. This hypothesis does not refer to the direct relationship between atherosclerosis and improved insulin sensitivity, but rather to the beneficial effects of improved insulin sensitivity on other risk factors in cardiovascular disease [95].

Jie Yang et al., in a study published in 2020, demonstrated that CR ameliorates various diseases, including cardiovascular disease, and effectively reduces atherosclerosis in apoE−/− mice, suggesting that CR is a powerful method and reproducible non-pharmacological therapy for managing atherosclerosis. To study the impact of CR on atherosclerosis development, apoE−/− mice were randomly divided into two groups, fed with a high-fat diet or CR for 16 weeks. Compared to mice in the high-fat-diet group, CR reduced body weight gain by approximately 12% and significantly decreased areas of atherosclerotic lesions from the aorta. A CR of 40% significantly reduced dietary fat content, which is associated with reduced atherosclerosis development in apoE−/− mice. However, the apoE−/− mice that benefited from a high-fat diet resulted in accelerated atherosclerosis progression. CR reduced body weight, improved lipid profiles, and inhibited the expression of inflammatory molecules in lesions [96].

A 6-year chronic CR reduced serum fasting plasma glucose levels and insulin low-density lipoprotein cholesterol (LDL-C), CRP, serum total cholesterol, PDGF-AB, blood pressure, BMI and carotid artery intima-media thickness [86,97]. Given the improvements that were observed in quality of life, studies show that, for CR to be effective and well-tolerated, it must not take place over a long period of time, as patients tend to give up CR. It is known that short-term CR is easier to include in clinical practice. Ellsworth DL et al. demonstrated through genomic analysis that the outcomes of short-term CR and long-term CR are similar [98].

CR is considered the most cost-effective intervention to reduce body weight and control risk factors for cardiovascular disease. To achieve weight loss and metabolic benefits, it is necessary to induce a negative energy balance by reducing caloric intake in obese subjects. This negative energy balance will have a favorable impact on the risk factors for cardiovascular diseases, particularly atherosclerosis [99]. CR reduces body weight, waist circumference by decreasing visceral fat, insulin levels and improves insulin sensitivity, and serum lipids, which will induce a reduction in proinflammatory adipokines, IL-6, IL-12, IL-18, and TNFα, etc., as well as an increase in anti-inflammatory adipokines, adiponectin and omentin, and lead to a significant reduction in oxidative stress [100,101]. Weight loss due to CR improves flow-mediated dilatation, which, in turn, significantly improves endothelial function in vitro in overweight adults [100].

CR has a complex molecular mechanism involving a reduction in insulin (IGF-1 pathway) and insulin-like signaling, mammalian target of rapamycin (mTOR) signaling kinase pathway, and an increase in the energy balance modulator sirtuin. From the same complex mechanism, a decrease is achieved in pro-inflammatory mediators, ROS production, and growth factors [87]. Sirtuins are responsible for the beneficial and longevity-promoting effects of CR in many animal species. Sirtuins have been shown to play an important role in physiological adaptation to CR with two distinct characteristics: resistance to oxidative stress and metabolic reprogramming to oxidative metabolism (required to access the highest possible energy from fuel sources) [102]. Sirtuins (especially sirtuin 1) are involved in many physiological effects, such as inflammation, aging, mitochondrial biogenesis apoptosis, and circadian clock control [103].

In this context, CR is an important clinical tool that reduces insulin signaling, and inflammation (NF-κB signaling and COX-2 expression-decreases pro-inflammatory adipokine levels), reduces angiogenic mediators, and pro-angiogenic leptin, and increases anti-angiogenic adiponectin levels [104,105]. CR has anti-inflammatory effects that contribute to endothelial function, which is directly related to the restoration of vascular tone, preservation of vascular wall integrity, regulation of angiogenesis, and hemostasis. It appears that the anti-inflammatory effect is not the only effect that CR exerts on the endothelium, in addition to the regulation of fibrinolysis, angiogenic factors, and the integrity of the basement membrane and extracellular matrix proteins [106]. CR influences angiogenesis by consistently decreasing circulating levels of the plasminogen activator inhibitor-1 (PAI-1), and matrix metalloproteinases (MMPs). It is shown that (PAI-1), u-PA, t-PA, and MMPs are deeply involved in angiogenesis [107]. CR leads to the increased expression of eNOS and the transcriptional factor nuclear erythroid factor 2-related factor (Nrf2), which activates the VEGF-dependent metabolic pathways and produces antioxidant stress proteins [88].

### 3.2. Fasting

Along with CR, fasting is proving to be an effective strategy to optimize health, reduce weight, and delay aging. Results from well-controlled investigations in animal and human experimental models have validated the method as being effective, but in certain situations, human subject compliance makes this infeasible. Fasting is defined as the absence of food or caloric drinks for periods ranging from 12 h to 16 h or, even more severely, 22 h. During the allowed hours, 2–3 meals are still eaten at one-hour intervals, and whole foods are still consumed. Fasting is associated with ketogenesis and promotes changes in metabolic pathways and cellular processes, such as lipolysis, autophagy, and stress resistance [108,109].

In 2015, Longo VD et al. concluded that intermittent fasting is less restrictive than traditional 40% CR, as daily caloric intake is similar to a short, strict CR. The authors highlighted the positive effects of intermittent fasting on cardiovascular health [110]. The most feasible form of fasting is intermittent fasting, including alternative fasting or fasting 2 days a week, for example. The most widely discussed forms of intermittent fasting are time-restricted feeding, in which individuals fast for 16 h–20 h a day; the 5:2 approach, meaning 2 non-consecutive days of energy restriction per week; or alternative days with total fasting.

Intermittent fasting has demonstrated its efficacy against the clinical progression of cardiovascular disease, inflammation, glucose intolerance, insulin resistance, obesity, and hypertension. The known mechanisms of intermittent fasting are the enhancement of cellular and molecular adaptive responses to stress, and here we mention endogenous antioxidant enzyme activity, mitochondrial function, proteostasis, and autophagy, as well as reductions in inflammation and oxidative stress [111].

In a clinical study of middle-aged individuals, Stekovic S. et al. demonstrated that 4-week intermittent fasting improved cardiovascular parameters, inflammatory markers, triiodothyronine and LDL levels, and significantly reduced trunk fat mass [112]. Whether intermittent fasting directly affects cardiovascular markers, or whether cardiovascular risks decrease secondary to weight loss, is debatable. However, a 3–24 week fast reduced the body weight of individuals enrolled in the studies, and the best results were obtained in individuals who had a low-calorie meal each day of fasting [113,114].

Klempel MC et al. compared alternative fasting and periodic fasting and demonstrated that alternative fasting is much more effective in reducing body weight [113]. However, both fasting strategies showed positive effects in reducing triglyceride levels, decreasing body weight [113,115].

Several studies show that weight loss from intermittent fasting is associated with decreased systolic and diastolic blood pressure, suggesting that both strategies prevent progression from prehypertension to hypertension [115]. Intermittent fasting has a positive effect on individuals with prediabetes by lowering blood glucose, but no changes were observed in healthy individuals. The reported evidence showed benefits in individuals who fasted on alternate days, and the insulin levels considerably decreased. Strong arguments have been made in favor of lowering insulin concentrations in studies in which subjects had severe CR, and lower insulin levels were associated with the severity of energy restriction [113,115].

### 3.3. Diet

Cardiovascular diseases are known to be the leading cause of death in developed countries due to the consumption of processed, high-carbohydrate, and high-fat foods. However, it has been observed that the incidence of cardiovascular diseases is much lower in Mediterranean countries, and this has mainly been attributed to dietary habits. In patients with coronary heart disease, following a Mediterranean diet is more effective in reducing the progression of atherosclerosis than following a low-fat diet. It appears that the consumption of extra virgin olive oil is a key element that could prevent the progression of atherosclerosis, as it is predominantly consumed in the Mediterranean diet [116,117].

The Mediterranean diet is rich in fish, vegetables, whole grains, fruit, nuts, and extra-virgin olive oil. In a systematic review on the effect of lifestyle and dietary changes in patients with coronary heart disease, Iestra JA et al. demonstrated that the mortality rate decreased in subjects who consumed extra-virgin olive oil compared to those who consumed other fats [118]. This could be explained by the improved endothelial function, reduced serum cholesterol and lower blood pressure found in individuals following the Mediterranean diet.

Estruch R et al. concluded that the Mediterranean diet, when supplemented with extra virgin olive oil and nuts, is the best-established dietary pattern to reduce cardiovascular parameters and lower apolipoprotein B [119]. Michael D. Shapiro and Sergio Fazioa demonstrated that cholesterol-rich lipoproteins containing apolipoprotein B are the most important causative agents of atherosclerotic cardiovascular disease. In the study, solid evidence was presented to support the theory low-density lipoproteins have an important function in the genesis of atherosclerosis [120].

Miguel A Martínez-González et al. hypothesized that the Mediterranean diet is the ideal nutritional model for cardiovascular health. The authors critically and systematically analyzed five meta-analyses published between 2014 and 2018 that included prospective observational cohorts and RCTs, totaling 45 prospective study reports. The evidence supporting the efficacy of the Mediterranean diet on cardiovascular health is numerous and consistent. The Mediterranean diet clinically significantly reduces rates of total cardiovascular disease, coronary heart disease, and ischemic stroke [121]. Dontas AS et al. have provided evidence that the Mediterranean diet provides protective benefits for cardiovascular health by being high in monounsaturated fats, low in saturated fats, and high in complex carbohydrates. Total fats account for about 40% of total energy intake and the monounsaturated to saturated fats ratio is around 2. Beta-carotene, vitamins C and E, various important minerals, and polyphenols are abundant in this diet, due to the high level of consumption of extra-virgin olive oil, vegetables, fresh fruits, and whole grains [122]. In The Seven Countries Study, Keys et al. demonstrated that coronary heart disease mortality is positively associated with saturated fat and inversely associated with monounsaturated fat. The Seven Countries Study was conducted on 16 cohorts of 12,763 middle-aged men who followed the Mediterranean diet. The Mediterranean diet improves the blood lipid profile (decreases triglycerides and low-density lipoprotein cholesterol) and decreases the risk of thrombosis, resulting in improved endothelial function [123,124].

Schwingshackl and Hoffmann demonstrated that the Mediterranean diet is effective in the prevention of cardiovascular disease due to its anti-inflammatory action and improved endothelial capacity [125].

**Figure 1:**
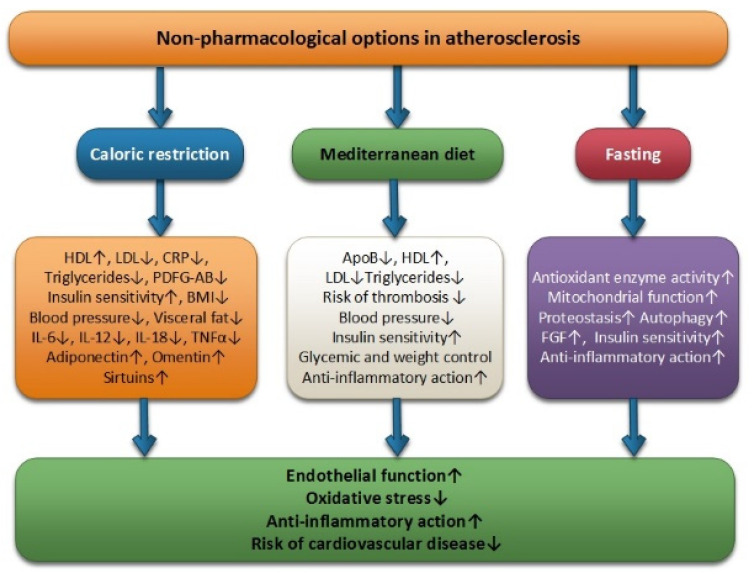
Non-pharmacological options in atherosclerosis: caloric restriction, Mediterranean diet and fasting.

Fasting, the Mediterranean diet, and CR can be considered useful clinical tools in the management of atherosclerotic cardiovascular disease by managing conventional risk factors. These non-pharmacological options are positively associated with increased endothelial function, anti-inflammatory effect, and reductions in low oxidative stress, thus reducing the risk of cardiovascular disease (Figure 1).

### 3.4. Physical Exercise

**Figure 2:**
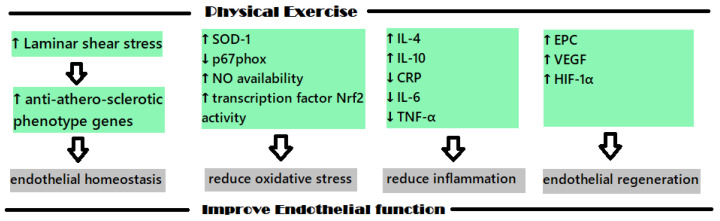
The main mechanisms involved in the protective cardiovascular effects of PE.

The protective impact that physical exercise (PE) has on the cardiovascular system has been studied in recent years with the intention of explaining the mechanisms involved; the increase in heat shock proteins, antioxidant enzymes and regulators of cardiac myocyte proliferation concentration seem to be the molecular and biochemical shifts that are involved [126]. Figure 2 shows the main mechanisms involved in the positive impact of PE on endothelial dysfunction.

The endothelium is a monolayer of endothelial cells that acts as an interface between the blood flow and the intima of blood vessels. As the endothelial cells are organized in to align with the direction of blood flow, they are directly affected by the physical forces induced by this flow. When sustain laminar shear stress (LSS) acts in vitro, an upregulation of the anti-athero-sclerotic phenotype genes is determined at the physiological levels, which seems to also manifest in vivo. These types of genes were identified using the GeneCalling method, and some representatives are as follows: intracellular adhesion molecule-1 (ICAM-1), eNOS, Cu/Zn superoxide dismutase (SOD), thrombomodulin, aldehyde dehydrogenase 6, SMAD6, heme oxygenase-1, cyclooxygenase 2, transforming growth factor (TGF)-b1 [127]. These genes that are related to LSS are associated with inflammation, oxidative stress and metabolism, apoptosis, cellular growth and differentiation, and seem to play an important role in the preservation of the endothelial homeostasis [127].

Studies conducted on animals have shown that PE has a positive impact on superoxide dismutase type 1 (SOD-1) when it is performed regularly as aerobic exercise training, and decreases the level of p67phox, which is a subunit of (NAD (P)H oxidase [127,128].

Other studies conducted on rabbits suffering from hypercholesterolemic femoral arteries show that PE restored the endothelium response to calcium and the endothelium-dependent relaxation [127,129]. In addition, another study shows that PE increases the eNOS expression of proteins [130].

In a review published in 2013, Golbidi and Laher discussed how PE can prevent and restore the endothelial dysfunction caused by aging, arguing that it increases NO availability through its antioxidant and anti-inflammatory role [131]. It is known that sustained PE leads to an improvement in antioxidant levels, which can be explained by the increase in oxidative stress levels that occur during PE. The fact that ROS increase as a result of this effort causes the organism to adapt to this type of stress, which translates to a better response to the repair mechanisms for oxidative damage, an increased resistance to oxidative stress and a decrease in the levels of oxidative damage [131].

For example, the transcription factor “nuclear factor (erythroid-derived 2)-like 2 (Nrf2)” plays an important role in PE’s positive effect on the endothelium dysfunction because it is linked to the organism’s fight against oxidative stress [131]. Nrf2 regulates the expressions of some antioxidants, such as NQO-1, glutathione-S-transferase, glutathione peroxidase, and HO-1 when located in the nucleus; however, in the absence of oxidative free radicals, it usually remains dormant in the cytoplasm of the cells [131,132].

During PE, a short-term inflammatory response appears, which is followed by a long-term anti-inflammatory adaptative response. This short-term inflammatory response correlates with an increase in the number of leukocytes, oxidants and C-reactive protein (CRP) level. When exercise is methodically performed, a decrease in the pro-inflammatory molecules level is noticed, and an increase in anti-inflammatory molecules levels is also noticed; substances such as IL-4 and IL-10 are produced and CRP, IL-6 and TNF-α levels decrease [131]. Regarding IL-6, this cytokine has both pro-inflammatory and anti-inflammatory effects; the anti-inflammatory effect is present when this molecule is secreted in the muscles and is related to the inhibitory effect is has on TNF-α, IL-10, IL-1ra and IL-1β [131].

Endothelial precursor cells (EPC) are important cells that are implicated in the regeneration of the endothelium and, depending on their number, they are positively associated with vascular function. These cells originate in the bone marrow and are circulating precursors of endothelial cells, but their circulating levels are usually small and, when injuries appear, the body has to mobilize them in higher levels with the aim of supporting endothelial repair [133]. Studies conducted in this field have revealed that physical effort can act as an impulse for the mobilization of EPC from bone marrow [134].

Ribeiro et al. conducted a study with the aim of assessing the impact that different intensity resistance exercise, performed one time, has on EPC levels over 24 h. All participants in this study were females (n = 38). Along with the determination of EPC levels, the underlying mechanisms for EPC mobilization by effort was assessed using vascular endothelial growth factor (VEGF), the angiogenic factors stromal-cell-derived factor 1 (SDF-1α), erythropoietin (EPO) and hypoxia-inducible factor 1-alpha (HIF-1α) [135]. After exercise, the number of EPCs increased, with the greatest increase occurring 6 h after effort was performed. This was also corelated with increasing levels of VEGF and HIF-1α. It was also observed that higher-intensity exercises were associated with a greater increase in EPC levels [135].

In a review published in 2021, Ferentinos et al. studied the impact of different forms of exercise on EPC levels in patients suffering from cardiovascular and metabolic diseases. Thirty-six trials were included in this study. The authors concluded that the diseases and type of exercise that was performed influenced EPC mobilization and that the regularly performed PE has an impact on the magnitude of EPC circulating levels [136]. For example, in patients suffering from chronic heart failure, after moderate- to high-intensity PE training, EPC levels showed an acute increase, and in patients suffering from ischemic or revascularized coronary artery disease maximal exercise tests, an acute increase in EPC levels was observed [136].

With regard to cardiovascular diseases, PE seems to play an important part in the prevention of this type of disease, and also in reducing the mortality risk. It has also been shown that inactivity and a sedentary lifestyle are related to a higher risk of CVD and mortality. PE causes an increase in cardiac output and blood flow [137]. The beneficial effects of PE seem to have greater impact in patients who have cardiovascular risk factors or suffer from cardiovascular diseases [131].

In 2014, Gu Qi et al. published two studies regarding the protective effect of physical exercise on the vascular system of rats, with a positive impact on aortic stiffening and endothelial dysfunction. The first study was conducted on 344 male rats, 3-month old and 23-month old males, divided into three groups: a sedentary group of young rats, sedentary group of old rats, and exercised-trained old rats. The third group performed chronic aerobic exercises on treadmills for 8 weeks. The efficacy of the exercise protocol was assessed by measuring citrate synthases in the soleus and gastrocnemius muscles [138]. The following determination was performed: measurement of collagen and elastin contents in aorta, aortic relaxation in response to acetylcholine and sodium nitroprusside, pulse wave velocity (PWV) measurement, measurement of malondialdehyde, measurement of aortic 3-nitrotyrosine, measurement of mitochondrial function of aortas, measurement of mitochondrial electron transport chain enzyme activity and measurement of mitochondrial electron transport chain enzyme activity [138]. The study showed an increase in collagen and elastin levels and a reduction in PWV in the group of rats exposed to PE, which is related to attenuated aortic stiffening. They also showed that PE can prevent aging-related ED, as shown by improvements in the endothelium-mediated vascular relaxation of aortas in response to acetylcholine. Another important aspect was that PE had a positive impact on oxidative stress and inflammation, as shown by a decrease in the formation of reactive oxygen species and mitochondrial swelling, which was related to the preservation of the aortic mitochondrial function [138]. The second study was conducted with the aim of assessing and understanding the mechanisms of the positive vasculature effect of PE. Two experiments were performed, and the finding was similar to the results of the previous study [139].

PE’s impact on the structure and function of the carotid was studied by Königstein, et al. In this study, 2893 adolescents and young adults were included, with ages between 14 and 28 years old; 49.6% of the participants were females. The efforts performed by each participant were assessed with the help of a questionnaire. Carotid intima-media thickness (cIMT) and stiffness (cS), which are early markers of atherosclerosis and vascular aging, were measured with the help of real-time B-mode ultrasound sequences with semi-automated edge-detection and automatic electrocardiogram-gated quality [140]. Atherosclerotic risk was assessed using the cumulative index for atherosclerotic risk (CV-R), which included the determination of mean blood pressure, HbA1c, triglycerides, body mass index and total/HDL-cholesterol ratio. The study results show a better cardiovascular profile for participants that declared high levels of exercise, but not for cS and cIMT. This can be due to the fact that the changes occurring in this age group are subtle [140].

Tomoto et al. studied the same theory in 70 patients suffering from amnestic mild cognitive impairment. Patients that participated in moderate-to-vigorous-intensity aerobic exercise training (AET) two years prior to the study, and patients that suffered from uncontrolled diabetes or hypertension or were obese, were excluded from the study. The participants were included in a 12-month program of moderate-to-vigorous AET or stretching-and-toning (SAT) interventions [141]. Forty-eight of the 77 participants completed one year of training, and the results showed that AET decreased carotid-stiffness index and cerebral blood flow (CBF) pulsatility, increased normalized CBF and improved peak oxygen uptake (VO2peak) [141].

The effects of PE on vascular function and other health measurements in pediatric patient survivors of oncology cerebral insults were studied by Long et al. In this 48-week study, 13 survivors aged between 16 and 23 were included. The exercise intervention lasted 24 weeks and different variables were assessed at the beginning of the study and at 24 and 48 weeks. The endothelial function was assessed using flow-mediated dilation (FMD). Other measurements were also performed: body composition, blood pressure, heart rate, muscular strength, aerobic capacity, anthropometry, muscular endurance and physical activity levels using accelerometers [142]. The results at baseline were compared with those obtained after 28 weeks of exercise intervention and FMD of the brachial artery suffered an increase, with shows that exercise has a positive impact on vascular function [142].

New studies are emerging because one of the main pathophysiological mechanisms of COVID-19 was shown to be endothelial dysfunction. One of these studies was conducted by Ambrosino et al. in 2022, and assessed the existing connection between cardiopulmonary exercise performance and endothelial dysfunction in patients suffering from COVID-19 [143]. The results of this study show that the disruptions that occurred in the endothelial barrier of the systemic and pulmonary circulation played an important role in the reduction in cardiopulmonary exercise testing performance, and new therapies (pharmacological and rehabilitation) that target the endothelial functions should be found [143].

In addition to the exercise’s important role in the normal maintenance of the vascular endothelium, it also has important anti-inflammatory and antioxidant roles. Physical exercise is an efficient clinical tool that limits chronic inflammation by increasing anti-inflammatory cytokines levels and limits pro-inflammatory cytokine by reducing oxidative stress [144].

The recommendations regarding moderate-intensity exercises are exercise for 30 min/day 5 days/week for most people. For people with diseases such as autonomic disorders, this training should be conducted under expert supervision.

### 3.5. Vagus Nerve Stimulation

From the previously discussed subjects, it appears that the pathophysiological mechanisms involved in the formation of atherosclerosis are excessive sympathetic stimulation, the inflammatory process, oxidative stress and endothelial dysfunction. Finding a way to simultaneously combat these factors would be ideal for the prevention of atherosclerosis. Vagus nerve stimulation could meet these criteria. Several non-pharmacological methods to activate the vagus nerve exist, such as the invasive and non-invasive electrical stimulation of the vagus nerve, and non-invasive stimulation of vagus nerve during different types of deep breathing or during heart rate variability biofeedback (HRVB) training.

Vagus nerve stimulation (VNS) can modulate the myocardial redox state to reduce oxidative stress [145]. VNS reduced protein oxidation in one study in mice with a myocardial infarction [146]. Using the cholinergic anti-inflammatory pathway, VNS could reduce cytokines levels with attenuation of the inflammatory process. This inhibitory pathway may be of interest to control the neural reflex of inflammation, with great potential in combating inflammatory diseases [147,148]. Several clinical studies have used implanted nerve stimulators to activate the vagus nerve, which have reported encouraging results in relieving chronic inflammation. In experimental models, it has been reported that interruption of the neuronal signal in the vagus nerve aggravates systemic inflammation [149,150,151,152].

The vagus nerve activity inhibits sympathetic activity [153]. The vagus nerve also induced increases in vasoactive intestinal peptide, which then increases coronary blood flow [154]. Hypoxia due to excessive sympathetic stimulation, oxidative stress and inflammation contributes to the formation of a vicious cycle that can be successfully combated by VNS [155].

VNS can be subcutaneous (sVNS) or transcutaneaous (tVNS). sVNS was approved for the treatment of refractory epilepsy and depression [156]. sVNS used bipolar electrodes tunneled under the skin, wrapped around the left vagus nerve in the neck, and connected to a pulse generator that is surgically implanted in the chest wall or axilla [157,158]. Less invasive VNS methods were developed, using electrode placement inside the internal jugular vein at spinal level C5–C7 (referred to as transvenous VNS) [159]. There is no evidence that transvenous VNS can decrease cytokine levels to date [159].

Invasive electrical stimulation of the vagus nerve is difficult andha s many risks, such as trauma to the vagus nerve, which can easily occur during surgical isolation, and suspension of the nerve on the electrode. The physiology of the nerve is significantly affected by compression maneuvers and stretching during manipulation. This causes physical stress on the nerve, which interferes with nerve function [160]. The electrodes used to stimulate the vagus nerve can be finely made of platinum-iridium, silver and tungsten-titanium. The most important characteristic of the electros is the integrity of the surface in contact with the vagus nerve and a stable load delivery to a high-quality, constant current voltage source converter [161,162].

Another important component of direct contact is that a non-toxic, efficient and stable interface must be created between the stimulation electrode and the nerve. The integrity of the electrode can be affected by various factors, such as long-term exposure to buffered saline, so it is mandatory to measure the electrical impedance at the interface during electrical stimulation [163].

The most common clinical use of VNS involves the surgical implantation of a commercially available programmable pulse generator device (NCP System; Cyberonics, Inc., Houston, TX, USA) [164]. The generator is subcutaneously implanted in the left upper chest or left axillary margin. The electrode lead is attached to the left middle cervical vagus nerve through a second incision in the left neck area. The leads wire is passed through a subcutaneous tunnel and attached to the pulse generator. Various surgical complications may occur, including wound infection and hoarseness as a result of temporary or permanent paralysis of the left vocal cord, which can be found in approximately 1% of patients.

To set the parameters of the stimulation generator and program the mode of operation, a laptop is needed. The transmission/programming system will be connected to this laptop, which will be placed on the patient’s skin, above the device. The parameters that can be set are as follows: charge current (electrical stimulus intensity, measured in milliamps (mA)), pulse width (electrical pulse duration, measured in microseconds), pulse frequency (measured in Hertz (Hz)), and on/off duty cycle (stimulus on and off time, measured in seconds or minutes). The initial settings for the four parameters can each be adjusted to optimize efficacy (for seizure control or other symptom control, depending on the indication) and tolerability [164]. The generator runs continuously, but patients can temporarily turn off VNS by holding a magnet over the device, and VNS can be turned on and off by the programmer. The lifetime of the pulse generator battery depends on the stimulus parameters and could be permanently replaced or removed by a simple surgical procedure.

A VNS device system (CardioFit System; BioControl Medical Ltd., Yehud, Israel) has been developed for the treatment of heart failure [165]. This programmable device is implanted in the right chest wall. This is connected to the right cervical vagus using a cuff designed to preferentially activate vagal efferent fibers (intended to affect cardiac function). The pacemaker detects the heart rate and stops at a predetermined bradycardia threshold. Current preclinical phase II studies suggest that chronic right cervical VNS is safe and effective for treating heart failure [166]. A similar VNS system (FitNeS System; BioControl Medical Ltd., Yehud, Israel) has been designed, with a cuff electrode that preferentially activates afferent fibers, which aims to minimize the typical VNS side effects related to efferent fiber stimulation. Left cervical VNS using this device was described in five patients with epilepsy, who showed some benefits and did not experience the side effects that are typical of VNS [167].

The least invasive tVNS methods use superficial stimulation of the vagus nerve through the skin, which can be applied to the anterolateral surface of the neck (cervical tVNS) or to the cymba conchae on the ear (auricle tVNS) [156]. Studies on the reduction in inflammation caused by tVNS are just beginning, but some are promising [168,169]. A recent study in 26 migraine patients receiving cervical tVNS (n = 14) or sham stimulation were performed over 2 months. Stimulation was self-administered bilaterally, twice daily for 120 s. Before and after 2 months of serum IL-1β, IL-6, IL-10, and TNFα were measured. After the 2-month period, serum IL-1β was higher in the sham control condition as compared to the tVNS group, and only mild relief was observed in migraine patients [168]. In another study, 20 healthy males and females were randomized to receive either nVNS or sham stimulation at 8.30 a.m., 12 p.m., and 6 p.m. An initial blood sample was taken at 8 a.m., and another blood sample was withdrawn 90 min and 24 h after the first stimulation session. The study highlights a significant decrease in the IL-1β, TNF, IL-8, macrophage inflammatory protein [MIP]-1α, and monocyte chemoattractant protein [MCP]-1 levels, which was observed in the nVNS group that was non-lipopolysaccharide (LPS)-stimulated after 24 h. The nVNS group showed a significant percent increase in LPS-stimulated IL-10 levels at the 24 h timepoint in comparison with the sham stimulation [169].

These studies revealed mixed results, but less invasive tVNS was allowed, which will improve our ability to test for the causal effects of VNS on inflammation. Given the novelty of VNS in humans for anti-inflammatory effects, there is no standardized method of treatment (administration methodology, duration, current levels) so the development of new guidelines for researchers and practitioners interested in vagus nerve modulation for inflammation control is needed. Future research using larger samples are needed to provide stronger evidence regarding the effect of tVNS on inflammation.

Furthermore, tVNS contributes to reduced activity in limbic brain regions [170], and was more recently found to increase activity in the anterior cingulate and the left prefrontal cortex [171]. These studies may suggest that tVNS can increase executive control and emotional regulation. Higher executive function can modulate risk factors such as smoking, unhealthy diets and sedentary behaviors, and can moderate the intention–behavior link between physical activity and dietary behavior [172].

Transcutaneous electrical nerve stimulation devices can also be used to deliver tVNS by placing contact electrodes in the cymba cup region. Patients can self-administer tVNS, which can be applied unilaterally or bilaterally (depending on the device system used), but there is no established clinical paradigm for how tVNS should be administered (i.e., stimulation parameters, duration and treatment frequency in each stimulation session, duration of treatment, etc.).

Another type of tVNS device (gammaCore; electroCore LLC, Basking Ridge, NJ, USA) has European approval for the prophylactic and acute treatment of cluster headache, migraine, persistent hemicranias and medication overuse headache. Therapy using gammaCore is delivered through a hand-held device with two flat stimulation contact surfaces that transmit a proprietary electrical signal in the vicinity of the vagus nerve. The device is placed on the neck over the vagus nerve in an area in which the subject’s pulse can be located. [173]. The intensity of the stimulation is controlled by the patient, and the application of the stimulation lasts for 90 s. Patients may experience headache relief when this is used as needed, but the device can be used several times a day to prevent headaches.

VNS studies have exponentially increased in recent years [174,175]. Expanding knowledge of the mechanisms of neural control of vascular inflammation, balance autonomic nervous system activities, reduce ROS/RNS will be important for the treatment of cardiovascular diseases such as atherosclerosis.

Another non-invasive method that stimulates the vagus nerve is paced vagal breathing, which is practiced during different types of physical activities, meditation, yoga, or during heartrate variability biofeedback (HRVB) training.

There is increasing evidence that yoga appears to have beneficial effects on the cardiovascular system through downregulation of the hypothalamic–pituitary–adrenal axis (HPA axis) and the SNS by increasing vagal activity and by improving baroreceptor sensitivity [176]. Yoga may restore the autonomic balance between SNS and PNS, by posterior hypothalamus inhibition, which generates a decrease in SNS activity [177].

Kiecolt-Glaser et al. (2014) found a reduction in the levels of IL-6, IL-1B, or TNF-α after 3 months of yoga training [178]. In a study, researchers found lower levels of transcription factor nuclear factor-κB, and increased levels of glucocorticoid receptor after yoga sessions. Additionally, soluble TNF receptor Type II remained stable in the yoga group, whereas, in the control group, it continued to increase, reflecting increasing inflammation [179]. Sarvottam et al. (2013) found decreased levels of plasma IL-6, increased levels of adiponectin, and an unchanged level of endothelin-1 among metabolically at-risk individuals after 10 days of yoga intervention [180].

HRVB is a non-invasive therapy training that aims to increase heartrate oscillations through real-time feedback and slow breathing training [181]. HRVB has a positive effect on psychological symptoms and increases wellbeing [182,183]. Lehrer et al. recently performed a systematic and meta-analytic review on the efficacy of HRVB and/or paced breathing (six breaths/min) and conclude that HRVB improves emotional and physical health and performance [184].

HRVB may have regulatory effects on the autonomic nervous system function. By enhancing vagal activity and reducing SNS activity, HRVB could represent a promising method for the management of a wide range of chronic diseases.

Evidence of biofeedback’s effects on inflammation is still limited. In a study by Lehrer et al. (2010), 11 healthy adults received an administration of endotoxin, and then the patients were randomly assigned to biofeedback training or control conditions. HRV parameters and serum cytokines (IL-6, IL-8, TNFα) were measured. Biofeedback training resulted in increases in time-domain measures of HRV, although no group differences were observed in cytokine levels [185]. Another study tested the effect of biofeedback training on airway inflammation. After 3 months, increases in resting RSA and reductions in eNO were observed in the biofeedback group, but not in controls [186]. HRVB significantly decreased systolic blood pressure and improved baroreflex sensitivity [187]. HRVB was associated with a reduced systolic blood pressure in response to exercise [188]. Thus, HRVB is a relatively simple, non-invasive technique that could be implemented in cardiac rehabilitation programs. Autonomic nervous system activity improvements represent one of the essential mechanisms through which HRV-biofeedback influences cardiovascular outcomes.

## 4. Summary and Future Directions

In this review, the recent scientific literature was carefully analyzed to demonstrate the existence of a close relationship between autonomic nervous system dysfunctions and atherosclerosis.

Atherosclerosis is no longer considered a simple lipid storage disorder but a systemic low-grade chronic inflammation, which is characterized by the persistence of a pro-inflammatory and a high oxidative state. Common cardiovascular risk factors, such as a high-saturated-fat diet, smoking, hypertension, hyperglycemia and insulin resistance, tend to produce chronic inflammation, which leads to endothelial dysfunction with impaired NO production, vasoconstriction and losses of the antithrombotic properties of the endothelium.

Psycho-emotional stress can cause dysfunction in both the autonomic and neuroendocrine nervous systems, which can lead to increased serum cortisol, catecholamine and an imbalance between SNS and PNS. Stress produces increased sympathetic activity, reduced vagal activity, and causes inflammation, oxidative stress and endothelial dysfunction. Ghiadoni et al. reported that acute mental stress induced SNS stimulation and transient endothelial dysfunction lasting up to 4 h, accompanied by increases in blood pressure, heart rate, and salivary cortisol [189]. Moreover, arterial stiffness in large artery was associated with increased SNS activity and impaired carotid baroreflex sensitivity [190]. Endothelial dysfunction could also impair vascular neurotransmission in tail arteries [191].

Psychosocial stress was associated with the upregulation of SNS and the increased proliferation of neutrophils and inflammatory monocytes in mice and humans [192,193]. Individuals with a higher tonic vagal output showed a lower expression of NF-κB transcription [194]. Following social stress, robust increases were found in circulating IL-1β and IL-6 [195]. Resting respiratory sinus arrhythmia was inversely related to increases in inflammatory cytokines, such as: IL-6, IL-8, IL-10, and TNFα [196]. Increases in cytokines were also inversely related to resting cardiac autonomic balance. Children with a higher resting cardiac autonomic balance, reflecting greater PNS, and less SNS modulation of the heart, exhibited reduced increases in cytokines [196].

A low resting HRV has been associated with greater increases in cytokines. Resting parasympathetic modulation of the heart, indexed through both high-frequency heart rate variability and low-frequency heart rate variability, and six circulating markers of inflammation, were evaluated in 836 adults [197]. Statistical analyses revealed inverse high-frequency associations with interleukin-6 (IL6), C-reactive protein (CRP), and fibrinogen [197]. The results suggest that the parasympathetic modulation of inflammation through the vagus nerve may act on specific inflammatory molecules [197].

The current understanding of the complex network that links the central and peripheral autonomic nervous system, hypothalamic–pituitary–adrenal axis, immune system, and the main biological systems provides a physiological explanation that links psycho-emotional stressors and social adversities to cardiovascular diseases [198].

## 5. Conclusions

The worldwide costs of cardiovascular diseases are enormous and the potential costs increase each year. Understanding the mechanism involved in atherosclerosis is a veritable key that opens the door to a proper therapeutic approach. Excessive sympathetic stimulation, inflammatory process, oxidative stress and endothelial dysfunction are the main mechanisms that contribute to the formation of atherosclerosis plaques. Finding a way to simultaneously combat these factors would be ideal in the prevention of atherosclerosis.

## Data Availability

Not applicable.
